# Endoscopic transorbital avenue to the skull base: Four-step conceptual analysis of the anatomic journey

**DOI:** 10.3389/fonc.2022.988131

**Published:** 2022-09-02

**Authors:** Giulia Guizzardi, Alberto Di Somma, Matteo de Notaris, Francesco Corrivetti, Juan Carlos Sánchez, Isam Alobid, Abel Ferres, Pedro Roldan, Luis Reyes, Joaquim Enseñat, Alberto Prats-Galino

**Affiliations:** ^1^ Laboratory of Surgical Neuroanatomy, Universitat de Barcelona, Barcelona, Spain; ^2^ Department of Neurological Surgery, Hospital Clínic de Barcelona, Barcelona, Spain; ^3^ Department of Neuroscience, Neurosurgery Operative Unit, “San Pio” Hospital, Benevento, Italy; ^4^ Laboratory of Neuroscience, European Biomedical Research Institute of Salerno (EBRIS) Foundation, European Biomedical Research Institute of Salerno, Salerno, Italy; ^5^ Clinic Institute of Ophthalmology (ICOF), Hospital Clínic de Barcelona, Barcelona, Spain; ^6^ Rhinology Unit and Smell Clinic, ENT Department, Hospital Clínic de Barcelona, Barcelona, Spain; ^7^ Servei de investigación en anatomía funcional del sistema nervioso, Institut d’Investigacions Biomèdiques August Pi i Sunyer (IDIBAPS), Barcelona, Spain

**Keywords:** endoscopic transorbital, endoscopic skull base surgery, endoscopic transorbital anatomy, skull base anatomy, surgical planning

## Abstract

**Background:**

In the last decades, skull base surgery had passed through an impressive evolution. The role of neuroanatomic research has been uppermost, and it has played a central role in the development of novel techniques directed to the skull base. Indeed, the deep and comprehensive study of skull base anatomy has been one of the keys of success of the endoscopic endonasal approach to the skull base. In the same way, dedicated efforts expended in the anatomic lab has been a powerful force for the growth of the endoscopic transorbital approach to the lateral skull base.

Therefore, in this conceptual paper, the main steps for the anatomic description of the endoscopic transorbital approach to the skull base have been detailed.

**Methods:**

The anatomic journey for the development of the endoscopic transorbital approach to the skull base has been analyzed, and four “conceptual” steps have been highlighted.

**Results:**

As neurosurgeons, the eyeball has always represented a respectful area: to become familiar with this complex and delicate anatomy, we started by examining the orbital anatomy on a dry skull (step 1). Hence, *step 1* is represented by a detailed bone study; *step 2* is centered on cadaveric dissection; *step 3* consists in 3D quantitative assessment of the novel endoscopic transorbital corridor; and finally, *step 4* is the translation of the preclinical data in the real surgical scenario by means of dedicated surgical planning.

**Conclusions:**

The conceptual analysis of the anatomic journey for the description of the endoscopic transorbital approach to the skull base resulted in four main methodological steps that should not be thought strictly consequential but rather interconnected. Indeed, such steps should evolve following the drives that can arise in each specific situation. In conclusion, the *four-step* anatomic rehearsal can be relevant for the description, diffusion, and development of a novel technique in order to facilitate the application of the endoscopic transorbital approach to the skull base in a real surgical scenario.

## Introduction

In the last decades, skull base surgery had passed through a remarkable evolution. Its development has been characterized by an evolving philosophy of minimally invasive attitude (1, 2). In the beginning, skull base surgery was performed through complex and extensive transcranial approaches, with a possibly high grade of complications and esthetic impairments (3–7). The advent of the endoscopic techniques has represented a revolution for the treatment of skull base lesions. First of all, the endoscopic endonasal route is nowadays a pillar of this kind of surgery and with its extended variants permits to access the entire skull base (8–12). In the last decades, to overcome the limits of the endonasal pathway to reach the lateral areas of the skull base, the transorbital route has recently been introduced with promising results (13–19). The role of neuroanatomic research has been uppermost, and it has played a central position during the expansion of these minimally invasive techniques directed to the skull base. Foremost, the deep and comprehensive study of skull base anatomy has been one of the keys of success of the endoscopic endonasal approach to the skull base (8, 10, 11, 20–22). Without this anatomical information that came from anatomical laboratory investigations, an efficient intraoperative orientation and a safe surgical limit would be hard to identify. As for the endonasal pathway, in the same way, dedicated efforts expended in the anatomic lab has been a powerful force for the growth of the endoscopic transorbital approach to the lateral skull base (23–29). Specifically, in our anatomic laboratory, our group has been focused, in the last decade, on the study of the application and technical variations necessary to develop the endoscopic transorbital route (25, 30–33). Thus, in this conceptual paper, we report the four main steps for the anatomic description of the endoscopic transorbital approach to the skull base which have been detailed and validated, according to our anatomic laboratory experience.

## Methods

The anatomic journey for the development of the endoscopic transorbital approach to the skull base has been analyzed. When facing with a new technique, especially an interesting “unusual” anatomical area, we always think about the anatomic fundamentals. To keep confidence with the anatomical region, in this case we started in the Laboratory of Surgical Neuroanatomy (LSNA) of the Human Anatomy and Embryology Unit, University of Barcelona (Barcelona, Spain), and all our works have ethical approval from the IRB of our University.

First of all, we identified the principal bone landmarks on a human dry skull and performed a bone drilling of the structures interested by the transorbital approach (i.e., lateral orbital wall, lesser sphenoid wing). Second of all, we performed the approach on various cadaveric specimens whose arterial and venous systems have been injected with red and blue latexes, respectively.

All specimens underwent a multi-slice helical computed tomography (CT) scan (SIEMENS Somatom go.Top software version VA30A-SP03) with 0.5-mm thick axial spiral sections and a 0° gantry angle, before and after dissection. In addition, the samples were submitted to high-field MRI (1.5 Tesla), to increase the accuracy of neurovascular and soft tissue structure characterization and neuronavigation software to allow the precise reconstruction of 3D models.

These data were collected and analyzed using Amira Visage Imaging (Amira Visage Imaging Inc., San Diego, California, USA), and a virtual 3D model of the dissected specimens was created; this quantitative analysis of the data permits us to estimate the necessary volume of bone removal to acquire satisfactory working angles during surgery. This analysis led to the study and evaluation of patients’ imaging with a huge amount of preclinical data, which helps in presurgical planning, to obtain a proper surgical corridor and a safer surgery.

Finally, we tried to identify the most important conceptual steps that characterized the entire process of the development and assimilation of the transorbital approach in our surgical routine, to share our experience and to make it reproducible.

## Results

**Figure 1:**
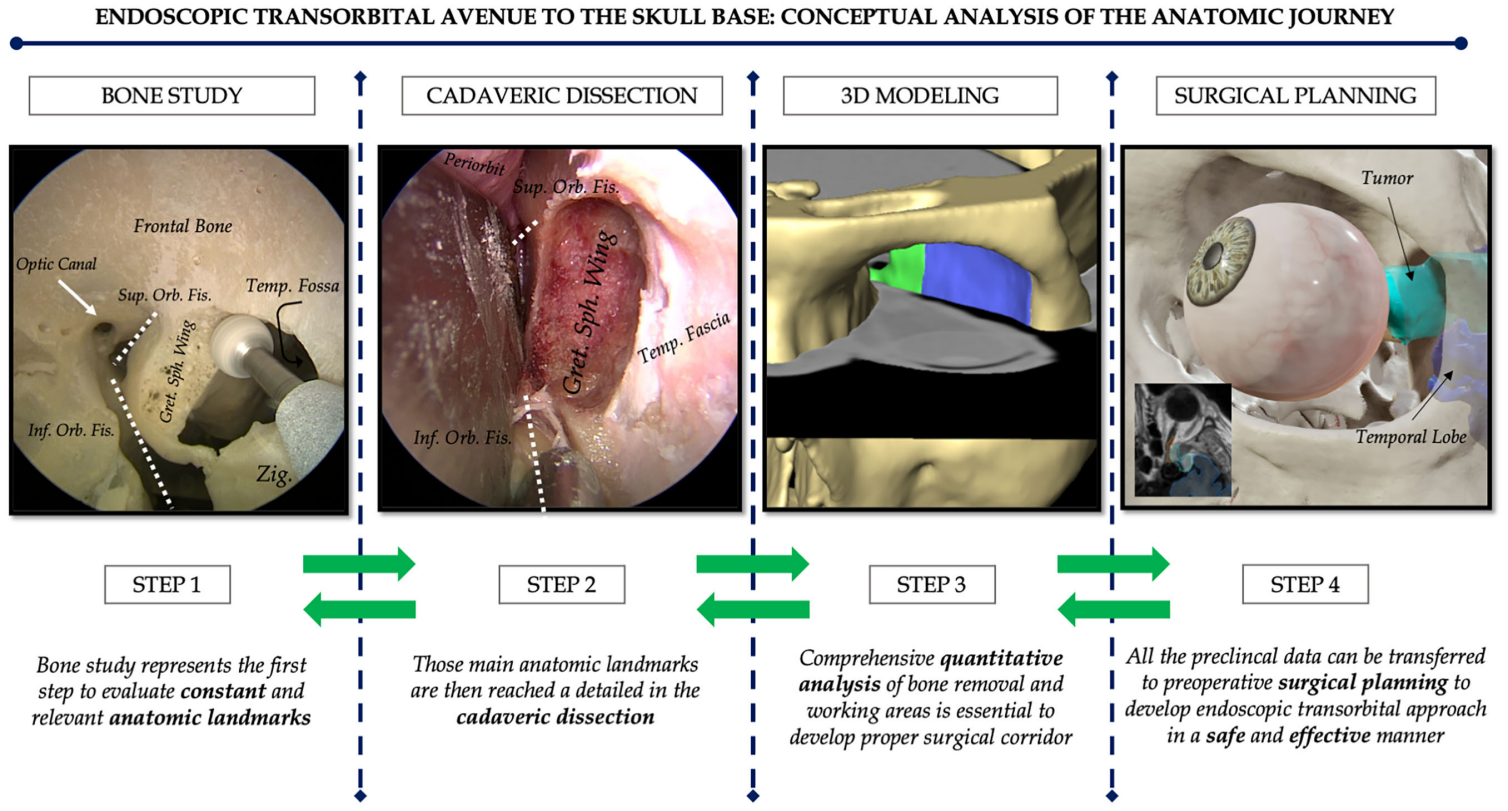
The four “conceptual” steps for the anatomic description of the endoscopic transorbital approach to the skull base. Step 1 is represented by a detailed bone study; step 2 is centered on the cadaveric dissection; step 3 consists in the 3D quantitative assessment of the novel endoscopic transorbital corridor; step 4 is the translation of the preclinical data in the real surgical scenario by means of dedicated surgical planning. Green arrows are shown in both directions to stress the concept that the anatomic rehearsal is not consequential (meaning from step 1 to step 4) but rather interconnected and bidirectional so that the steps should evolve not in chronological order, but following the drives that can arise in each specific situation.

As a result, four principal steps were described, and detailed in the following (**Figure 1**).

### Step 1

We started by examining the orbital anatomy on a dry skull: the lateral orbital rim, the superior and inferior orbital fissure, the greater sphenoid wing, and the middle fossa floor landmarks. First, in the orbit, the lateral orbital rim and the superior and inferior orbital fissure permit us to maintain orientation. The superior orbital fissure is delimited by the lesser and greater wings of the sphenoid. The inferior orbital fissure is bounded by the greater sphenoid wing and the maxilla and orbital process of the palatine bone.

Within the lesser wing, the optic canal is excavated. The drilling in the upper portion of the lesser sphenoid wing, in an upward direction from the orbit, permits to unlock the anterior cranial fossa and to reach and possibly remove the anterior clinoid process.

**Figure 2:**
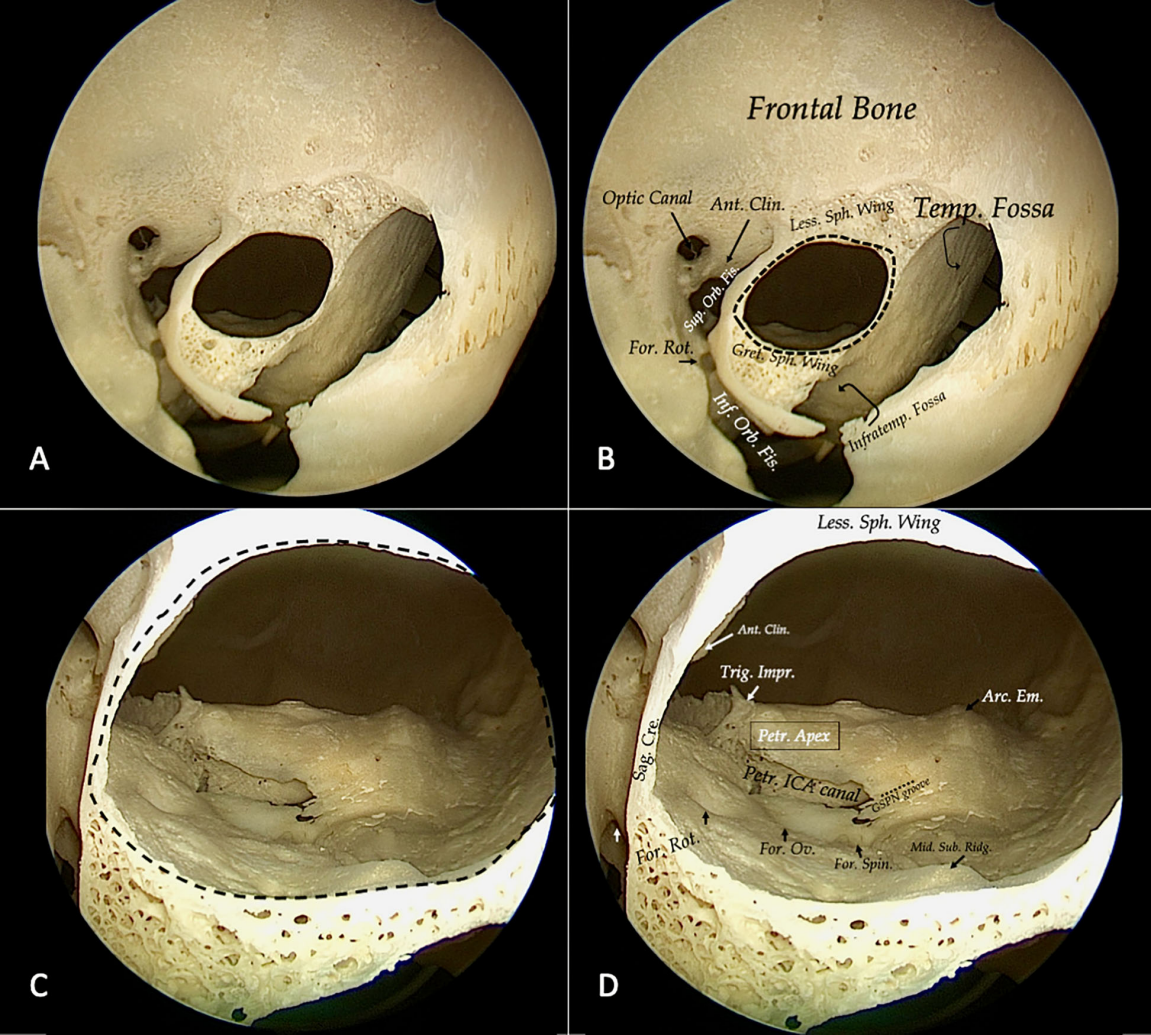
Step 1 is always represented by a detailed bone study; in this case, we use the left orbit of a dry skull. After a first superficial drilling of the sphenoid bone, orbital bone landmarks could be identified **(A, B)**. It is crucial to collocate lettering to identify clearly bone structures: optic canal, the superior (Sup. Orb. Fis.) and inferior orbital fissure (Inf. Orb. Fis.), the foramen rotundum (For. Rot.), the anterior clinoid process (Ant. Clin.), lesser (Less Sph. Wing) and greater sphenoid wing (Great. Sph. Wing), temporal fossa (Temp. Fossa), and the infratemporal fossa (Infratemp. Fossa). Afterward, we proceed to identify middle fossa bone landmarks (black dotted line). Medially the sagittal crest (Sag. Cre.) and on the floor foramen rotundum (For. Rot.) ovale (For. Ov.) y spinosum (For. Spin.), the middle subtemporal ridge (mid. Sub. Ridg.), posteriorly GSPN groove and the canal of petrous internal carotid artery, laterally the arcuate eminence (Arc. Em), and medially the trigeminal impression (Trig. Imp.) and the petrous apex (Petr. Apex) **(C, D)**.

The removal of the greater sphenoid wing permits us to unlock the middle fossa floor. Here we can identify multiple important bone landmarks. Laterally we can visualize the midsubtemporal ridge, which is a bony prominence in form of a crest, systematically seen in the mid-lateral part of the floor and the foramen spinosum which contain the middle meningeal artery. Posteriorly the arcuate eminence protrudes from the petrous segment of the temporal bone. Here we can identify, as posterior limit of bone removal, the carotid canal containing the GSPN and the petrous segment of the ICA. More medially is the foramen ovale, for the mandibular branch and the foramen rotundum for the maxillary branch of the trigeminal nerve, the latter representing the medial limit of our approach. The foramen rotundum is opened by drilling of the middle fossa floor (**Figure 2**).

Going forward, the removal of the floor of the middle fossa opens access to the posterior aspect of the temporal bone and is possible to remove by drilling a portion of the petrous apex and work in the posterior cranial fossa. After bone analysis, a study is necessary to confirm the results with cadaveric dissection.

### Step 2

**Figure 3:**
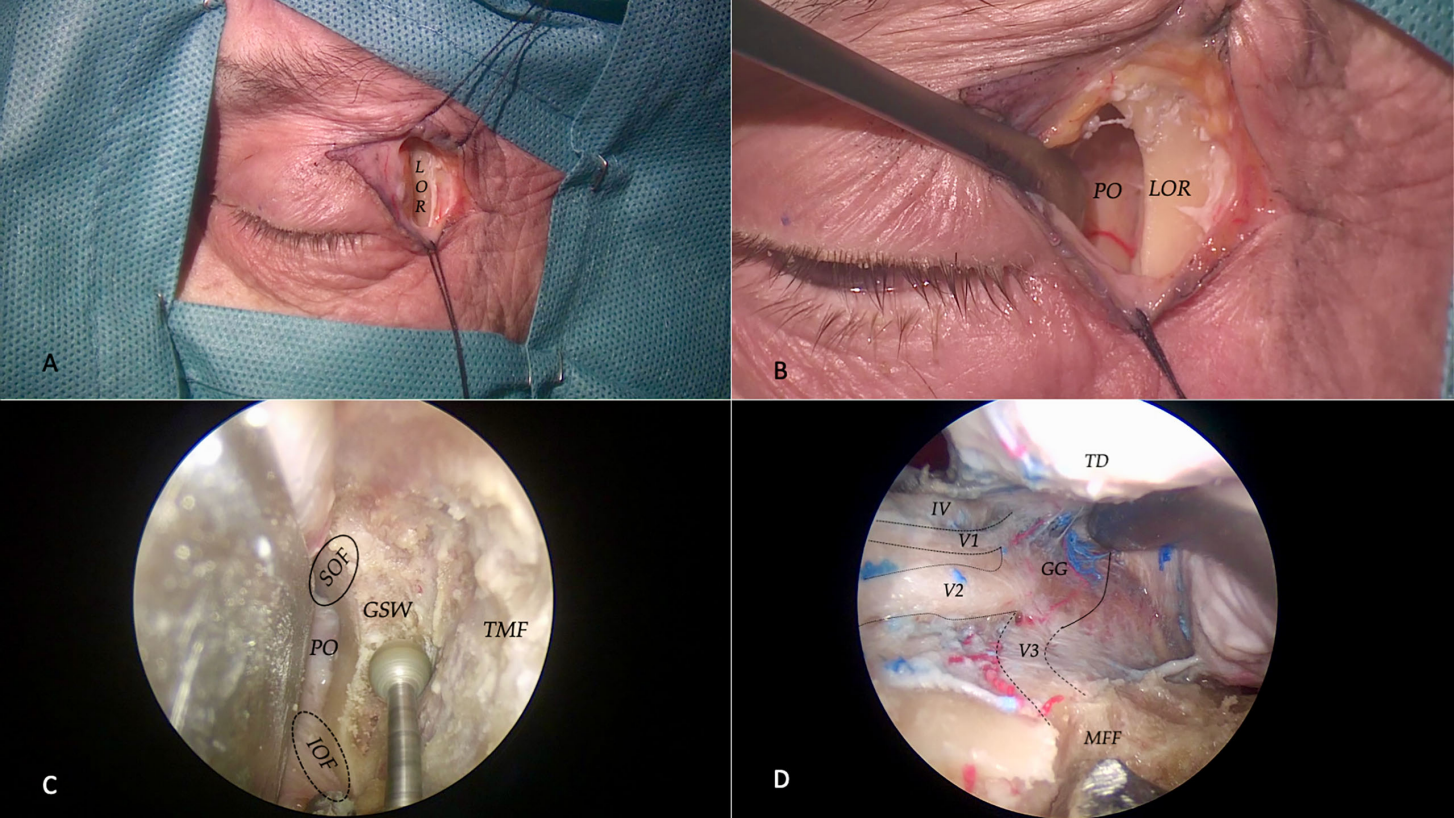
Step 2 is centered on cadaveric dissection. For a superior eyelid transorbital approach, a curvilinear incision is made, and after muscle dissection, the lateral orbital rim (LOR) is identified **(A)**. At this point, the periosteum is incised and a subperiosteal dissection is realized to mobilize the periorbita (PO) and ocular contents medially **(B)**. At this point, the endoscope is inserted to drill out the greater sphenoid wing (GSW), to expose, laterally, the temporalis muscle fascia (TMF), and posteriorly the temporal dura. Our medial limits are represented by superior (SOF) and inferior orbital fissures (IOF) **(C)**. Once having completed the drilling of the middle fossa floor (MFF), the temporal dura (TD) could be dissected and elevated **(D)**: this is the typic transorbital view of the middle fossa anatomy with the three trigeminals branches (V1, V2, and V3), the trochlear nerve (IV), and the gasserian ganglion (GG).

Subsequently, *step 2* consists in recognizing the main bone anatomic landmark in cadaveric dissection, and afterward labeling all the main neurovascular structures that can be encountered in the anterior middle and posterior fossa. Considering a lateral superior eyelid approach, constant bone anatomic landmarks have been described. After skin incision and muscle dissection, the superolateral orbital rim was seen down to the frontozygomatic suture. The periosteum was then dissected toward the orbit, and a surgical plane between the periosteum and the periorbita was created. At this point, the orbital orifices for zygomaticofacial and zygomaticotemporal branches are identified: these arteries are useful for proper identification of the inferior orbital fissure (IOF), which represents the most medial, inferior, and ventral landmarks of the approach to the middle fossa. Isolation of the superior orbital fissure (SOF), in the most posterior (that is, deep from a transorbital perspective) and lateral aspect of the surgical corridor, is essential for adequate orientation. To access the anterior cranial fossa, the lesser sphenoid wing should be removed; at this point, in an extradural fashion, it is possible to remove the anterior clinoid process. Removing the body of the zygoma is mandatory to obtain working space and bone removal by drilling the greater sphenoid wing to unlock the middle fossa dura. When elevating the dura of the temporal lobe, the foramen rotundum, the petrous internal carotid artery canal, the arcuate eminence, the foramen ovale, the foramen spinosum, the midsubtemporal ridge, and the sagittal crest of the sphenoid bone represent important anatomical landmarks of the middle fossa floor from a transorbital perspective (33). The three trigeminal branches and the gasserian ganglion are constant references in the middle cranial fossa anatomy (**Figure 3**). Finally, if the middle fossa floor is removed posteriorly, it is possible to access the petrous apex.

### Step 3

Successively, *step 3* embodies the quantification of the main anatomic targets that can be reached *via* the endoscopic transorbital superior eyelid approach. These data are essential to compare results with other studies and with other existing and alternative surgical techniques and, moreover, to assess objectively the feasibility of the approach: the evaluation of adequate exposure and working angle obtained with a volume of safe bone removal is crucial when analyzing a novel technique.

**Figure 4:**
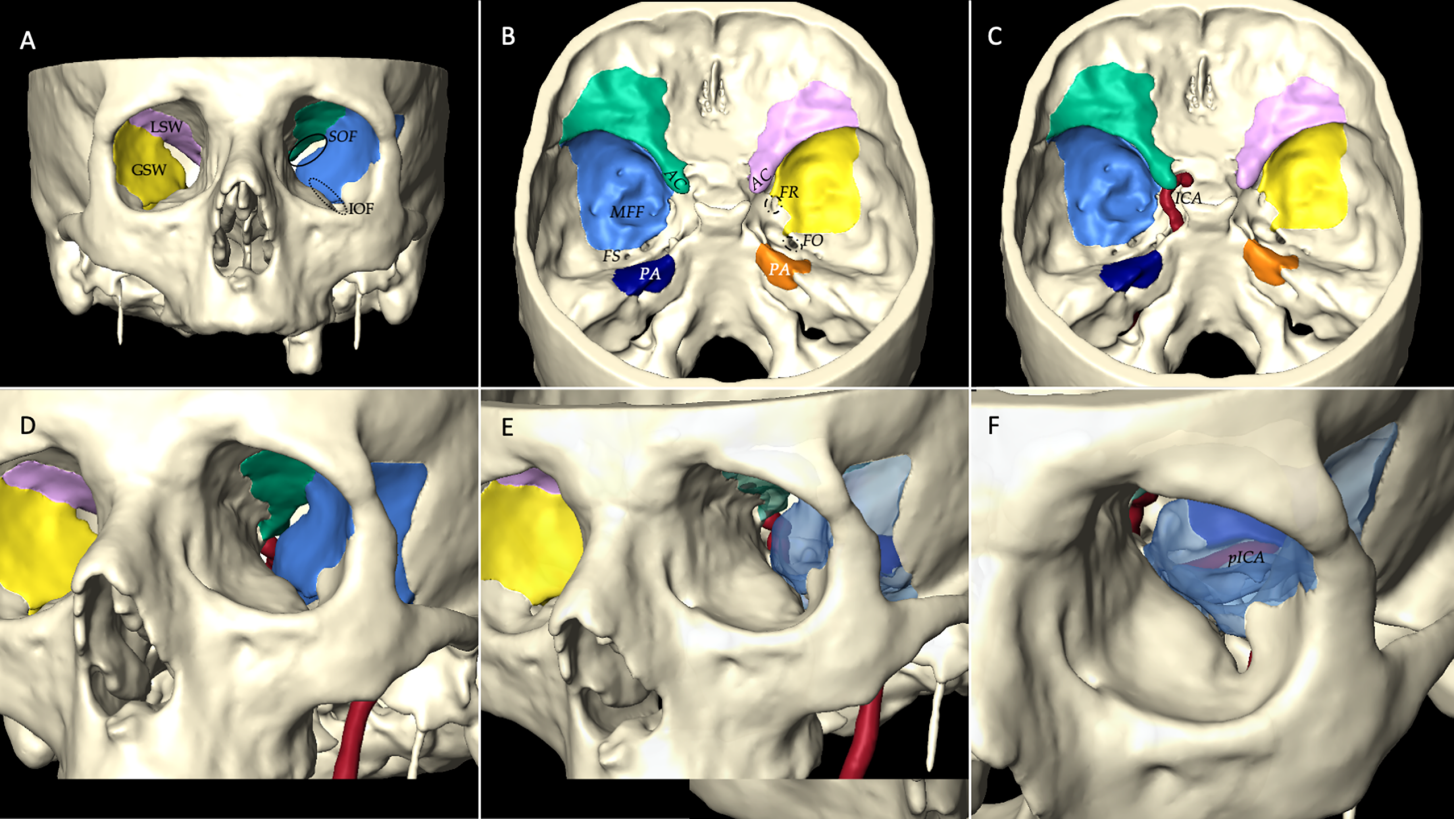
Step 3 consists of 3D quantitative and qualitative assessments of the post-dissection data. To perform this analysis, a virtual 3D model of the dissections is created using Amira Visage Imaging (Amira Visage Imaging Inc., San Diego, California, USA). This is an example of a 3D reconstruction model with bony and neurovascular structure reproduction. Specifically, in this case we calculated the volume of bone removal necessary to access the anterior (green in the left and rose in the right side), middle (light blue in the left and yellow in the right side), and posterior fossa (dark blue in the left and orange in the right side) from a transorbital endoscopic route: with an intraorbital perspective **(A)** and an intracranial perspective **(B)**. With Amira software, it is possible to reconstruct the course of the left internal carotid artery (ICA) **(C)**, to observe its position and location from a transorbital perspective **(D)**; furthermore, if we remove the bone drilled out with the transorbital route, and with different angulations **(E, F)** we can observe and identify the petrous segment of the internal carotid artery (pICA). LSW, lesser sphenoid wing; GSW, greater sphenoid wing; IOF, inferior orbital fissure; SOF, superior orbital fissure; AC, anterior clinoid process; MFF, middle fossa floor; PA, petrous apex; FS, foramen spinosum; FR, foramen rotundum; FO, foramen ovale.

For the quantitative analysis, a virtual 3D model of the dissections was created using Amira Visage Imaging (Amira Visage Imaging Inc., San Diego, California, USA). Bony and neurovascular structures were delimited in the MRI and in the CT scans, and these pertinent structures were then transferred to the Amira Workstation, which provided advanced instruments for measurement and quantification (**Figure 4**). Specifically, it permits us to calculate the volume of bone removal necessary to access the skull base, the working areas, and surgical freedom obtained with our approach.

### Step 4

**Figure 5:**
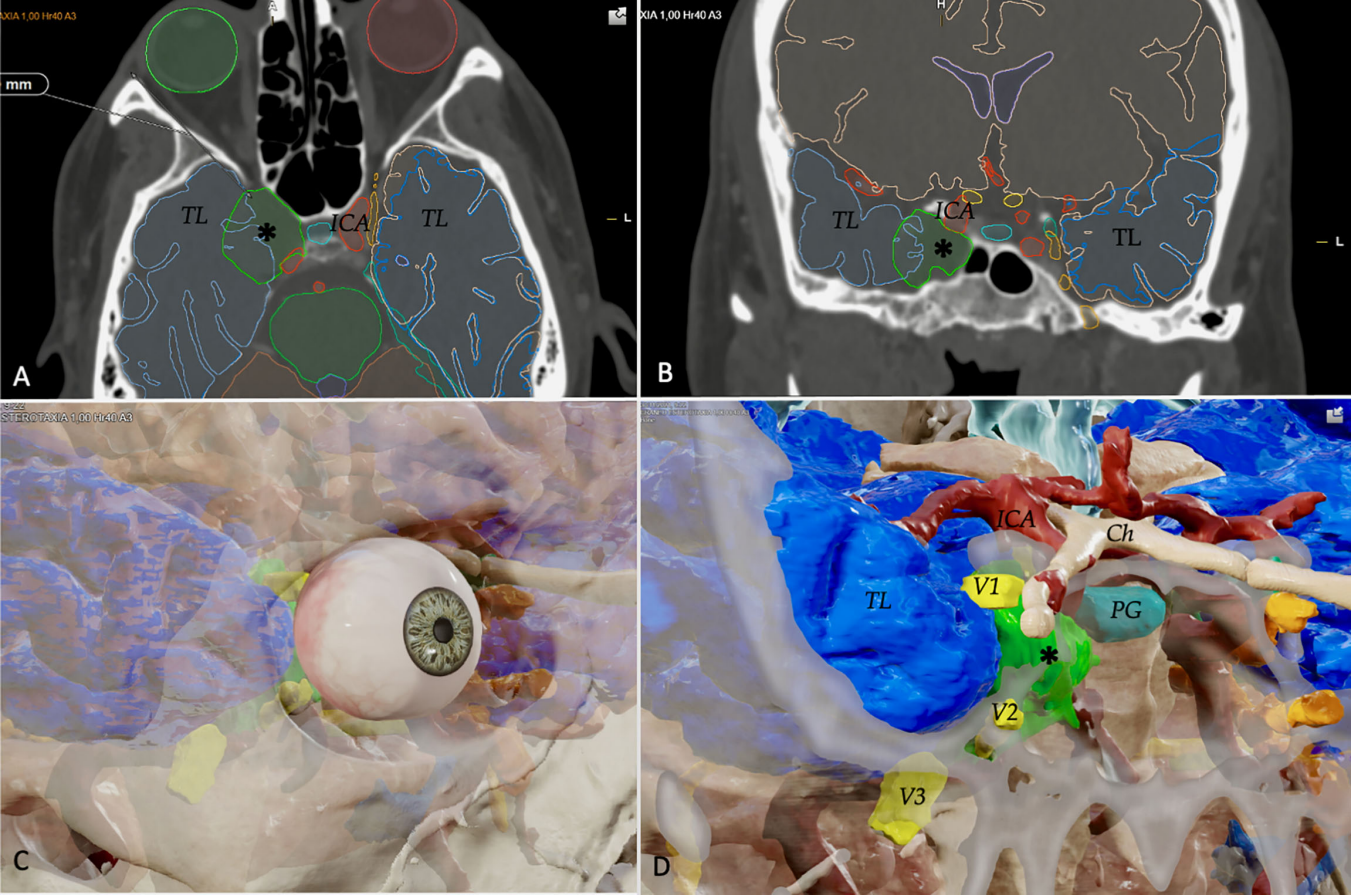
In Step 4, all preclinical data can be transferred to preoperative surgical planning to develop endoscopic transorbital approach. This is an example of a 3D model of a patient’s MRI with a cavernous sinus hemangioma, using the Brainlab (Brainlab Curve, Feldkirchen, Germany) navigation system to evaluate the presurgical planning. The system could highlight all the relevant neurovascular structures in the preoperative image in the axial **(A)** and coronal planes **(B)**: for example, the temporal lobes (TL), the tumor (black asterisk), and the internal carotid artery (ICA). From the MRI, it is possible to create a 3D model of the patient, to determine, basing on our anatomical data and studies, the feasibility and potential efficacy of the endoscopic transorbital approach in this case **(C, D)**. The location of the tumor (black asterisk) and other neurovascular, from a transorbital perspective, could be clearly seen: the three trigeminal branches (V1, V2, V3), the temporal lobe (TL), the internal carotid artery (ICA), the optic chiasm (Ch), and the pituitary gland (PG).

Finally, in *step 4*, all these preclinical data can be transferred to preoperative surgical planning to develop an endoscopic transorbital approach in a safe and effective manner. Step 4 is the last and relevant gait before entering in the operating room. In this case, we usually create a 3D model of a patient’s MRI, using the Brainlab (Brainlab Curve, Feldkirchen, Germany) navigation system to evaluate the presurgical planning (**Figure 5**). Using the quantitative data acquired from our anatomical study is possible to assess the feasibility of the transorbital approach for each patient.

## Discussion

At the beginning, the transorbital approach was reserved to intraocular pathologies and suffered an underestimation (34). Nevertheless, in the last years it has gone through a huge development, which made it popular in the skull base surgery community. Firstly, it was widely studied in anatomical settings (35–38) and preliminary surgical cases have been published (39–41). Our group has been deeply involved in this evolution process, becoming one of the reference centers in the use of the endoscopic transorbital approach in our country (25). Considering our positive experience, we decided to share the process that made this possible, from the initial ideas to the first surgical cases.

For neurosurgeons, the eyeball has always represented a respectful area: to become familiar with this complex and delicate anatomy, we started by examining the orbital anatomy on a dry skull, *step 1*. This initial step is crucial to identifying anatomical bone landmarks and how they are encountered from an endoscopic transorbital perspective (33, 37). As previously said, the identification of SOF and IOF is crucial for surgical orientation in the orbital phase. The optic canal remains medially outside the transorbital approach view. With the removal of the greater sphenoid bone, the middle fossa floor is visualized.

After bone characterization, we skip to the cadaveric dissection, *step 2*. Here is when the surgeon first intends to perform the procedure and is faced with technical nuances and challenges. A debated question is the management of the lateral orbital rim: many authors prefer to realize a lateral orbitotomy (42, 43). In our group, instead of performing a craniectomy, we realize a drilling of the inner part of the zygoma; due to our bone and anatomical studies, the space achieved with this maneuver is sufficient to permit a favorable working angle. Another important obstacle encountered to proceed in the transorbital approach and to access the posterior cranial fossa is the middle fossa floor. Due to its conformation, this bony structure limits the maneuverability when working posteromedially to the gasserian ganglion, trying to reach the petrous apex. With our dissections, we established a safe volume of bone removal (6.49 ± 0.80 cm^3^) that could be safely drilled without neurovascular damages and that are sufficient to access the posterior cranial fossa.

With the post-dissection data, we move to *step 3*, the quantitative analysis. At this moment, with the help of imaging software, we can create 3D models of our dissection. Volumes and areas could be calculated and compared. The angle of attack could be estimated and evaluated. At this point, the efficacy and feasibility of the technique are evaluated.

Finally, in *step 4* all our preclinical data and knowledge are transferred to the presurgical planning in clinical practice. This allows us to estimate the role of the endoscopic transorbital approach in each patient: we can predict which percentage of a lesion could be removed through a transorbital route—if the transorbital path alone would be sufficient or is better to consider the additional approach (“multiportal” fashion) (30, 35). We strongly believe that our four-step process would be extremely useful for transorbital technique diffusion and reproduction, leading to an improvement of patients’ care.

## Conclusions

The conceptual analysis of the anatomic journey for the description of the endoscopic transorbital approach to the skull base resulted in four main methodological steps that should not be thought strictly consequential but rather interconnected so that the steps should evolve following the drives that can arise in each specific situation. Hence, the four-step anatomic rehearsal can be relevant for the description, diffusion, and development of a novel technique in order to facilitate the application of the endoscopic transorbital approach to the skull base in a real surgical scenario.

## Data availability statement

The original contributions presented in the study are included in the article/supplementary material. Further inquiries can be directed to the corresponding author.

## Author contributions

GG, ADS, and AP-G contributed to conception and design of the study. GG organized the database. GG and ADS wrote the first draft of the manuscript. All the other authors suggested ideas and data due to their specialties. All authors contributed to manuscript final version, read, and approved the submitted version.

## Funding

This study has been funded by Instituto de Salud Carlos III (ISCIII) through the project PI19/00592 and cofunded by the European Union; it has also been funded by the “Fundació La Marató de TV3” (Reg. 95/210; Codi projecte 201914).

## Conflict of interest

The remaining authors declare that the research was conducted in the absence of any commercial or financial relationships that could be construed as a potential conflict of interest.

The handling editor KM declared a past co-authorship with the authors AD, MN, and JE.

## Publisher’s note

All claims expressed in this article are solely those of the authors and do not necessarily represent those of their affiliated organizations, or those of the publisher, the editors and the reviewers. Any product that may be evaluated in this article, or claim that may be made by its manufacturer, is not guaranteed or endorsed by the publisher.

## References

[1] Martinez-Perez R Requena LC Carrau RL Prevedello DM. Modern endoscopic skull base neurosurgery. J Neurooncol (2021) 151:461–75. doi: 10.1007/s11060-020-03610-9 33611712

[2] Arbolay OL González JG González RH Gálvez YH. Extended endoscopic endonasal approach to the skull base. Minim Invasive Neurosurg (2009) 52:114–8. doi: 10.1055/s-0028-1119414 19650013

[3] Saraceno G Agosti E Qiu J Buffoli B Ferrari M Raffetti E. Quantitative anatomical comparison of anterior, anterolateral and lateral, microsurgical and endoscopic approaches to the middle cranial fossa. World Neurosurg (2020) 134:e682–730. doi: 10.1016/j.wneu.2019.10.178 31731015

[4] Tomio R Horiguchi T Borghei-Razavi H Tamura R Yoshida K Kawase T. Anterior transpetrosal approach: experiences in 274 cases over 33 years. technical variations, operated patients, and approach-related complications. J Neurosurg (2021) 136(2):413–42. doi: 10.3171/2020.12.JNS204010. 34388716

[5] Kawase T Toya S Shiobara R Mine T. Transpetrosal approach for aneurysms of the lower basilar artery. J Neurosurg (1985) 63:857–61. doi: 10.3171/jns.1985.63.6.0857 4056899

[6] Kawase T Shiobara R Toya S. Anterior transpetrosal-transtentorial approach for sphenopetroclival meningiomas: surgical method and results in 10 patients. Neurosurgery (1991) 28:869–75; discussion 875-6. doi: 10.1097/00006123-199106000-00014 2067611

[7] Tripathi M Deo RC Suri A Srivastav V Baby B Kumar S. Quantitative analysis of the kawase versus the modified dolenc-kawase approach for middle cranial fossa lesions with variable anteroposterior extension. J Neurosurg (2015) 123:14–22. doi: 10.3171/2015.2.JNS132876 25839921

[8] Gardner PA Prevedello DM Kassam AB Snyderman CH Carrau RL Mintz AH. The evolution of the endonasal approach for craniopharyngiomas. J Neurosurg (2008) 108:1043–7. doi: 10.3171/JNS/2008/108/5/1043 18447729

[9] de Lara D Ditzel Filho LF Prevedello DM Carrau RL Kasemsiri P Otto BA. Endonasal endoscopic approaches to the paramedian skull base. World Neurosurg (2014) 82:S121–9. doi: 10.1016/j.wneu.2014.07.036 25496623

[10] Schwartz TH Fraser JF Brown S Tabaee A Kacker A Anand VK. Endoscopic cranial base surgery: classification of operative approaches. Neurosurgery (2008) 62:991–1002; discussion 1002-5. doi: 10.1227/01.neu.0000325861.06832.06 18580797

[11] Kassam AB Prevedello DM Carrau RL Snyderman CH Thomas A Gardner P. Endoscopic endonasal skull base surgery: analysis of complications in the authors' initial 800 patients. J Neurosurg (2011) 114:1544–68. doi: 10.3171/2010.10.JNS09406 21166570

[12] Zanation AM Snyderman CH Carrau RL Gardner PA Prevedello DM Kassam AB. Endoscopic endonasal surgery for petrous apex lesions. Laryngoscope (2009) 119:19–25. doi: 10.1002/lary.20027 19117306

[13] Kong DS Young SM Hong CK Kim YD Hong SD Choi JW. Clinical and ophthalmological outcome of endoscopic transorbital surgery for cranioorbital tumors. J Neurosurg (2018) 131:667–75. doi: 10.3171/2018.3.JNS173233 30215555

[14] Yoo J Park HH Yun IS Hong CK. Clinical applications of the endoscopic transorbital approach for various lesions. Acta Neurochir (Wien) (2021) 163(8):2269–77. doi: 10.1007/s00701-020-04694-y 33394139

[15] Park HH Roh TH Choi S Yoo J Kim WH Jung IH. Endoscopic transorbital approach to mesial temporal lobe for intra-axial lesions: Cadaveric study and case series (SevEN-008). Oper Neurosurg (Hagerstown) (2021) 21(6):E506–15. doi: 10.14791/btrt.2022.10.F-1299 34528091

[16] Lin BJ Ju DT Hsu TH Chung TT Liu WH Hueng DY. Endoscopic transorbital approach to anterolateral skull base through inferior orbital fissure: a cadaveric study. Acta Neurochir (Wien) (2019) 161:1919–29. doi: 10.1007/s00701-019-03993-3 31256277

[17] Schwartz TH Henderson F Di Somma A Kong DS De Notaris M Enseñat J. Endoscopic transorbital surgery: Another leap of faith? World Neurosurg (2022) 159:54–5. doi: 10.1016/j.wneu.2021.12.081 35007891

[18] Locatelli D Restelli F Alfiero T Campione A Pozzi F Balbi S. The role of the transorbital superior eyelid approach in the management of selected spheno-orbital meningiomas: In-depth analysis of indications, technique, and outcomes from the study of a cohort of 35 patients. J Neurol Surg B Skull Base (2022) 83:145–58. doi: 10.1055/s-0040-1718914 PMC9010142 35433179

[19] Chibbaro S Ganau M Scibilia A Todeschi J Zaed I Bozzi MT. Endoscopic transorbital approaches to anterior and middle cranial fossa: Exploring the potentialities of a modified lateral retrocanthal approach. World Neurosurg (2021) 150:e74–80. doi: 10.1016/j.wneu.2021.02.095 33647487

[20] de Divitiis O D'avella E De Notaris M Di Somma A De Rosa A Solari D. The (R)evolution of anatomy. World Neurosurg (2019) 127:710–35. doi: 10.1016/j.wneu.2019.03.050 31266133

[21] Cappabianca P Cavallo LM Solari D Esposito F. Endoscopic endonasal transsphenoidal approach to pituitary adenomas. J Neurosurg (2015) 122:473–4. doi: 10.3171/2014.8.JNS141716 25495741

[22] Solari D Chiaramonte C Di Somma A Dell'aversana Orabona G De Notaris M Angileri FF. Endoscopic anatomy of the skull base explored through the nose. World Neurosurg (2014) 82:S164–70. doi: 10.1016/j.wneu.2014.08.005 25496629

[23] Moe KS Bergeron CM Ellenbogen RG. Transorbital neuroendoscopic surgery. Neurosurgery (2010) 67:ons16–28. doi: 10.1227/01.NEU.0000373431.08464.43 20679952

[24] Lee MH Hong SD Woo KI Kim YD Choi JW Seol HJ. Endoscopic endonasal versus transorbital surgery for middle cranial fossa tumors: Comparison of clinical outcomes based on surgical corridors. World Neurosurg (2019) 122:e1491–504. doi: 10.1016/j.wneu.2018.11.090 30468930

[25] Di Somma A Sanchez España JC Alobid I Enseñat J. Endoscopic superior eyelid transorbital approach: how I do it. Acta Neurochir (Wien) (2022) 164(7):1953–59. doi: 10.1007/s00701-022-05177-y 35275271

[26] Di Somma A Andaluz N Cavallo LM De Notaris M Dallan I Solari D. Endoscopic transorbital superior eyelid approach: anatomical study from a neurosurgical perspective. J Neurosurg (2018) 129:1203–16. doi: 10.3171/2017.4.JNS162749 29243982

[27] López CB Di Somma A Cepeda S Arrese I Sarabia R Agustín JH. Extradural anterior clinoidectomy through endoscopic transorbital approach: laboratory investigation for surgical perspective. Acta Neurochir (Wien) (2021) 163(8):2177–88. doi: 10.1007/s00701-021-04896-y 34110491

[28] Dallan I Di Somma A Prats-Galino A Solari D Alobid I Turri-Zanoni M. Endoscopic transorbital route to the cavernous sinus through the meningo-orbital band: a descriptive anatomical study. J Neurosurg (2017) 127:622–9. doi: 10.3171/2016.8.JNS16465 27858571

[29] Zoia C Bongetta D Gaetani P. Endoscopic transorbital surgery for spheno-orbital lesions: how I do it. Acta Neurochir (Wien) (2018) 160:1231–3. doi: 10.1007/s00701-018-3529-5 29651750

[30] Di Somma A Langdon C De Notaris M Reyes L Ortiz-Perez S Alobid I. Combined and simultaneous endoscopic endonasal and transorbital surgery for a meckel's cave schwannoma: technical nuances of a mini-invasive, multiportal approach. J Neurosurg (2020) 134(6):1836–45. doi: 10.3171/2020.4.JNS20707 32650309

[31] Di Somma A Torales J Cavallo LM Pineda J Solari D Gerardi RM. Defining the lateral limits of the endoscopic endonasal transtuberculum transplanum approach: anatomical study with pertinent quantitative analysis. J Neurosurg (2018) 130:848–60. doi: 10.3171/2017.9.JNS171406 29676691

[32] De Rosa A Pineda J Cavallo LM Di Somma A Romano A Topczewski TE. Endoscopic endo- and extra-orbital corridors for spheno-orbital region: anatomic study with illustrative case. Acta Neurochir (Wien) (2019) 161:1633–46. doi: 10.1007/s00701-019-03939-9 31175456

[33] Corrivetti F De Notaris M Di Somma A Dallan I Enseñat J Topczewski T. "Sagittal crest": Definition, stepwise dissection, and clinical implications from a transorbital perspective. Oper Neurosurg (Hagerstown) (2022) 22(5):e206–12. doi: 10.1016/j.bas.2021.100594 35239519

[34] Houlihan LM Belykh E Zhao X O'sullivan MGJ Preul MC. From krönlein, through madness, to a useful modern surgery: the journey of the transorbital corridor to enter the neurosurgical armamentarium. J Neurosurg (2021) 5:1–10. doi: 10.3171/2020.8.JNS201251 33545682

[35] Di Somma A Guizzardi G Valls Cusiné C Hoyos J Ferres A Topczewski TE. Combined endoscopic endonasal and transorbital approach to skull base tumors: a systematic literature review. J Neurosurg Sci (2021) 65:000‒000. doi: 10.23736/S0390-5616.21.05401-1 34342198

[36] Di Somma A Cavallo LM De Notaris M Solari D Topczewski TE Bernal-Sprekelsen M. Endoscopic endonasal medial-to-lateral and transorbital lateral-to-medial optic nerve decompression: an anatomical study with surgical implications. J Neurosurg (2017) 127:199–208. doi: 10.3171/2016.8.JNS16566 27791520

[37] Di Somma A Andaluz N Cavallo LM Topczewski TE Frio F Gerardi RM. Endoscopic transorbital route to the petrous apex: a feasibility anatomic study. Acta Neurochir (Wien) (2018) 160:707–20. doi: 10.1007/s00701-017-3448-x 29288394

[38] Caklili M Emengen A Cabuk B Anik I Ceylan S. Endoscopic transorbital approach to the cavernous sinus lateral compartment (Anatomical cadaver study). Turk Neurosurg (2021) 31:813–9. doi: 10.5137/1019-5149.JTN.34972-21.2 34374985

[39] Park HH Hong SD Kim YH Hong CK Woo KI Yun IS. Endoscopic transorbital and endonasal approach for trigeminal schwannomas: a retrospective multicenter analysis (KOSEN-005). J Neurosurg (2020) 133:467–76. doi: 10.3171/2019.3.JNS19492 31226689

[40] Kim EH Yoo J Jung IH Oh JW Kim JS Yoon JS. Endoscopic transorbital approach to the insular region: cadaveric feasibility study and clinical application (SevEN-005). J Neurosurg (2021), 1–9. doi: 10.3171/2020.8.JNS202255 33482646

[41] Kong DS Kim YH Hong CK. Optimal indications and limitations of endoscopic transorbital superior eyelid surgery for spheno-orbital meningiomas. J Neurosurg (2020) 134:1472–9. doi: 10.3171/2020.3.JNS20297 32502989

[42] Ulutas M Çinar K Dogan I Secer M Isik S Aksoy K. Lateral transorbital approach: an alternative microsurgical route for supratentorial cerebral aneurysms. J Neurosurg (2019) 29:1–12. doi: 10.3171/2019.9.JNS191683 31783357

[43] Lim J Sung KS Kim W Yoo J Jung IH Choi S. Extended endoscopic transorbital approach with superior-lateral orbital rim osteotomy: cadaveric feasibility study and clinical implications (SevEN-007). J Neurosurg (2021) 12:1–14. doi: 10.3171/2021.7.JNS21996 34767525

